# A Global Health Research Checklist for clinicians

**DOI:** 10.1186/s12245-018-0176-9

**Published:** 2018-04-19

**Authors:** Rasha D. Sawaya, Kristen A. Breslin, Eiman Abdulrahman, Jennifer I. Chapman, Dafina M. Good, Lili Moran, Paul C. Mullan, Oluwakemi Badaki-Makun

**Affiliations:** 1 Children’s National Health System, The George Washington University School of Medicine and Health Sciences, 111, Michigan Ave, NW, Washington, DC, 20010 USA; 2 Division of Emergency Medicine, Children’s National Medical Center, 111, Michigan Ave, NW, Washington, DC, 20010 USA; 3 Department of Emergency Medicine, Children’s Hospital of The King’s Daughters, 601 Children’s Lane, Norfolk, VA 23507 USA; 4 Department of Pediatrics, Division of Emergency Medicine, Johns Hopkins University School of Medicine, 1800 Orleans St, Baltimore, MD 21287 USA; 5 Division of Emergency Medicine, The American University of Beirut Medical Center, PO box 11-0236, Riad El-Solh, Beirut, 1107 2020 Lebanon

## Abstract

Global health research has become a priority in most international medical projects. However, it is a difficult endeavor, especially for a busy clinician. Navigating the ethics, methods, and local partnerships is essential yet daunting.

To date, there are no guidelines published to help clinicians initiate and complete successful global health research projects. This Global Health Research Checklist was developed to be used by clinicians or other health professionals for developing, implementing, and completing a successful research project in an international and often low-resource setting. It consists of five sections: Objective, Methodology, Institutional Review Board and Ethics, Culture and partnerships, and Logistics. We used individual experiences and published literature to develop and emphasize the key concepts. The checklist was trialed in two workshops and adjusted based on participants’ feedback.

## Background

Discussions surrounding global health research priorities, methods, ethics, and governance have increased in recent decades following reports highlighting disparities in global research resource allocation, with an emphasis on local research capacity building, respect for local innovation, and global research priority setting [1–3].

In parallel, clinicians are increasingly interested in global health work as indicated by the increasing number of training programs with global health rotations or tracks and global health education publications [4–6]. In academic settings, clinicians aim to marry their global health clinical interests with academic research in order to increase their impact in their partner communities as well as their academic footprint, thereby developing an academic niche. However, performing research in a different country can be a daunting proposition for the busy clinician with limited time and funding, and maintaining such a research career may be difficult [7, 8].

To date, there are no guidelines published to specifically help clinicians initiate and complete successful global health research projects. We define a research project as successful if it reached publication and had a meaningful impact on the local community.

Therefore, we endeavored to develop a Global Health Research Checklist to be used by clinicians as a structure for developing, implementing, and completing a successful research project in an international and often low-resource setting.

The Global Pediatric Emergency Medicine Group (gPEM Group) at Children’s National Health Systems, George Washington University is a group of pediatric emergency medicine physicians with clinical and research experience in global health in various settings. Eight of us used a team approach based on our individual experiences to delineate the sections of the checklist. We used published literature to emphasize the key concepts as described below in detail. The checklist was trialed in two workshops including the Pediatric Academic Society meeting, Washington DC, and was adjusted based on participants’ feedback which included changes in structure, number of sections, examples, and content flow.

This checklist consists of five sections that can be tackled in any order (Table 1):

1. Objective
2. Methodology
3. Institutional Review Board and Ethics
4. Culture and partnerships
5. Logistics

**Table 1:** Global health research checklist for clinicians

Global Health Research Checklist
Objective ○ Needs assessment ○ Study specific aim ○ Global health/local community impact ○ *Is this section feasible?*
Methodology ○ Chose your GH research method ○ *Is this section feasible?*
Institutional Review Board and Ethics ○ Assess the ethical standards of the study ○ Obtain IRB approval from your home institution and local study site ○ *Is this section feasible?*
Culture and partnership(s) ○ Understand how the local culture and community influences your study or vice versa ○ Identify local partners ○ Define the roles in the collaboration ○ *Is this section feasible?*
Logistics ○ Timeline ○ Funding ○ International travel preparedness ○ *Is this section feasible?*

## The five sections of the checklist

Two successful research projects [9–11] illustrate key concepts of the checklist and will be referred to in the discussion below (Table 2).

**Table 2:** Case illustrations

Case one: “Pediatric Preparedness of Lebanese Emergency Departments” [9]	Case two: “Improving triage in a Botswana Emergency Department”. [10, 11]
Study summary: A nationwide survey of all Lebanese hospitals with Emergency Departments (ED) that care for children highlighted that care was provided by a variety of physicians, most without any specific pediatric, pediatric critical care, or pediatric emergency care training. Study checklist key points: • *Objective:* The goal of the principal investigator, RS, was to understand the precarious state of pediatric emergency medicine (PEM) in Lebanon in order to further its development. Specifically, she aimed to describe the EDs of hospitals that cared for children. • *Methodology:* A written survey of all Lebanese hospitals with EDs that care for children. • *Culture and partnership:* A partnership with an emergency medicine national leader was initially established via relationships that RS already had in place. Discussions between RS and the local partner lead to the specific aim. The participants’ roles in the project were delineated beforehand and authorship credits assigned. The key roles of the local partner included identifying a local research assistant, the wording of the survey in order to facilitate understanding by Lebanese physicians, and helping approach the different hospitals in Lebanon. Culturally, Lebanese respond better to personal contact; therefore, speaking the local languages and establishing and maintaining relationships were key in identifying a local partner as well as recruiting hospitals. • *Institutional Review Board and Ethics*: RS obtained Institutional Review Board (IRB) approval from the US home institution and from the local partners’ institution. • *Logistics:* Time and funding were all from personal resources. Study impact: A key result of the study, that unspecialized physicians care for acutely ill and injured children, identified areas for potential intervention. In response to these results, RS partnered with local physicians to create a PEM track in the following Lebanese Emergency Medicine Conference and is now developing a PEM curriculum for the first four-year Emergency Medicine residency program in Lebanon.	Study summary: A quality improvement project team adapted a regionally tested triage system, the South African Triage Scale (SATS) and renamed it the Princess Marina Hospital Accident & Emergency Triage Scale (PATS). Overall, over-triage rates and under-triage rates showed significant improvements, as PATS was more predictive of inpatient admission, Intensive Care Unit admission, and death in the ED than the prior triage system. Study checklist key points: • *Objective:* The Princess Marina Hospital Accident & Emergency (PMH A&E) leadership approached and partnered with the principal investigator, PM, to improve their triage system. • *Methodology:* Using the adapted SATS allowed, a SATS team of trainers, that was available in the region, to assist in the training of trainers within the PMH A&E group. • *Culture and Partnerships:* PM spent 2 years in Botswana working with physicians, developing partnerships and gaining familiarity with the local medical system and culture. A memorandum of understanding (MOU) was established between the senior hospital leadership in Botswana and PM’s sponsoring US institution. The role of PM in the project was to travel a few times per year from his home institution to organize the local staff in Botswana to design the project, build up local capacity to continue to manage it, and ensure its sustainability. • *Institutional Review Board and Ethics:* All of the data collected and analyzed received approval from the IRBs of PMH, the Botswana Ministry of Health, the University of Botswana, and PM’s US home institution. • *Logistics:* PM was able to secure funding for his travel from his home institution, the SATS trainers used their own funds to travel as an investment in their region’s healthcare, and the limited funding required for the rest of the project activities all came from resources within PMH which had a vested interest in improving its triage system and outcomes. Study impact: Developing this adapted triage system (PATS) fostered collaboration between two African countries as well as a US partner, and it promoted higher quality care for children with emergencies at the PMH A&E. This triage system is in year 5 as of 2014.

### Objective

As with any sound research project, the first step is defining the research question and objectives. Generally, the project will grow out of personal interests or, preferably in global health, the needs of the local partner. A review of the literature will help identify work already done in the specified field as well as people who have worked in the area of research and the country of interest. Many professional organizations have interest groups to connect with those working in similar areas in global health [12]. Ideally, in global health settings where the territory and the system may be unfamiliar, the investigator should identify both published and unpublished studies and learn about successes and roadblocks previous investigators faced. Most importantly, the on-site partners must be involved and invited to give feedback on the value and acceptability of the project, and a formal needs assessment can be a valuable tool.

For example, in case one, RS initially aimed to study pre-hospital care but in response to the local partner’s needs, changed to study the emergency department.

Next, the investigator, together with on-site collaborators, should formulate specific objectives from the potential broad research questions. This may be a testable hypothesis, such as a new triage system will have less over-triage than the previous system (case two), or a may be a specific aim describing the training of providers caring for children in local emergency departments (case one).

Finally, the investigator is encouraged to consider the potential impact of the study being planned. These include answers to the following questions: Why is this question worth investigating? What are the potential implications on health outcomes of individual and of the community? Is there a possible impact beyond the population studied? What are the implications for the health resource utilization for the hospital or ministry of health? Will the implementation of this study drain the system or contribute to it?

### Methodology

While any study design may be applied to global health research, the methodology selected will depend on the available data sources, ethical considerations, and resource limitations. A structured planning tool, such as a logic model [13], will help ensure all necessary resources and desired outputs are considered and will provide a map of the project to share with collaborators. Table 3 lists examples of study designs with special considerations for a global health setting.

**Table 3:** Examples of study design: advantages and pitfalls in a global health setting

Study design	Examples	Considerations
Experimental designs	Clinical trial Educational intervention Quality/process improvement	• For clinical trials, there must be reasonable uncertainty about whether the intervention or standard of care is better (equipoise) [36] • Educational and quality improvement projects may allow comparison of the same group before and after • Outcomes from educational interventions can be knowledge, attitudes, or behaviors
Observational designs [37]	Prospective cohort Retrospective cohort Case-control Descriptive epidemiology	• Review of existing records from a retrospective cohort or case-control requires reliable clinical or administrative records • Surveys and interview tools should be either validated tools from the published literature or carefully designed and reviewed [38] • Prospective data collection may require more time and personnel
Qualitative design [39]	Interviews	• May generate new ideas for further testing
Pilot study	Small-size project to assess feasibility	• Identifies potential problems prior to larger-scale study [40]

One priority is defining the study population. In a global setting, the local team’s input is invaluable to help understand the population and setting the investigator will be working in and how it will impact the study population.

Finally, the outcome specifies exactly what the study will measure. For example, to evaluate a new triage system, the investigator may want to measure how well the assigned triage level compares to the eventual disposition of the patients (case two). One could also measure staff satisfaction, length of stay, or in-hospital mortality. Using mortality as a measure can potentially show the importance of an intervention, but it can be difficult to demonstrate an impact and may require a larger sample size.

### Institutional review boards and ethics

Significant discussion regarding the ethics of research in developing versus developed countries has occurred in recent decades, leading to the elaboration of guidelines specific to research in international settings [14–16]. These highlight the need to consider local cultures, economic capabilities, population needs, the local team’s right to innovation, and self-governance as well as long-term benefits and sustainability to the community studied [17, 18].

The principle of respect for persons emphasizes the individual’s right to self-determination and requires protection of those with a lower capacity for self-determination (e.g., children) [19]. Informed consent is an integral part of this principle and one that often causes complications in research implementation. For instance, in some societies, it is traditional and acceptable for the husband or another male relative to make decisions for a woman [14]. In others, the consent of community elders or senior family members must be sought before individual consent is obtained, if it is to be obtained at all [15]. Finally, illiteracy and cultural perceptions of western medicine may prove to be significant barriers to obtaining informed consent [20]. Discussions with local partners early on will be invaluable in understanding cultural implications of the proposed study and adjusting study design accordingly.

The principle of beneficence centers on non-maleficence and maximizing benefits while minimizing risk [19]. One controversy arising from the implementation of this principle for research in developing countries is the question of “standard of care,” i.e., should research studies in developing countries be required to provide the best available therapies (often standard of care in developed countries) as controls or should the standard of care in the local setting (frequently no or minimal therapy) be used? Such dilemmas have occurred frequently in published literature [20–22].

The principle of justice is exemplified by fairness of distribution. It requires appropriate selection of subjects and requires that the population in which the research study is being performed directly reap the benefits of the study [19]. Case 2 illustrates this principle: in Botswana, the host hospital was left with a new triage system uniquely designed specifically for it [10, 11].

To protect research subjects and follow the basic ethical principles outlined above, there is a requirement that proposed research studies be reviewed by independent bodies based on ethical merit and scientific validity [14, 15]. In principle, research should be held to high standards regardless of location. Generally, studies in developing countries involving cross-national collaborations require review by Institutional Review Boards (IRBs) (or Research Ethics Committees) in both the developed and developing countries [14]. Identifying IRBs in developing countries may be difficult. If not available at the host institution, IRBs may be available at nearby large educational institutions/universities. In addition, local or national Ministries of Health could provide such services. Finally, if after a good faith effort to locate an IRB, but one does not exist at all in the country of interest, guidelines exist to assist in establishing one specifically for the study in question (Table 4).

**Table 4:** Institutional review board resources

Office for Human Research Protection (OHRP) [41] Institutional Review Board Guidebook [42] Institutional Review Board Identification: Office for Human Research Protections (OHRP) Database for Registered IORGs & IRBs, Approved FWAs, and Documents Received in Last 60 Days [43]	

### Culture and partnership(s)

Culture is pervasive in life, impacting every aspect of human behavior. The undertaking of a project in a different country where language, customs, religion, economic, and political climates all vary requires significant preparation [23]. A transparent relationship with a focus on humility and respect for the local partner’s culture is essential [24]. Sometimes, even a well thought out project may be affected by an unanticipated cultural misunderstanding [25–29].

In case one, RS had a rich understanding of the local culture given that she had lived there for many years. In creating the hospital surveys, since Arabic and English are the two languages utilized by most physicians, the team decided not to translate the survey in French, another main language in Lebanon. It was during the analysis that the word “resident” was noted to mean “someone who works in the hospital” for the physicians not trained in the US (or similar) medical system. This underscores the fact that even when a researcher has extensive knowledge about a culture, he or she may be from a different social class, ethnicity, or religion and therefore have a different understanding on certain issues and may not be aware of all the cultural variations within one nation.

In another unpublished example by LM, a project investigating the understanding of child abuse in Ghana was unsuccessful in getting local IRB approval because it was deemed to be too sensitive a topic with too many cultural implications for the local community.

**Fig. 1:**
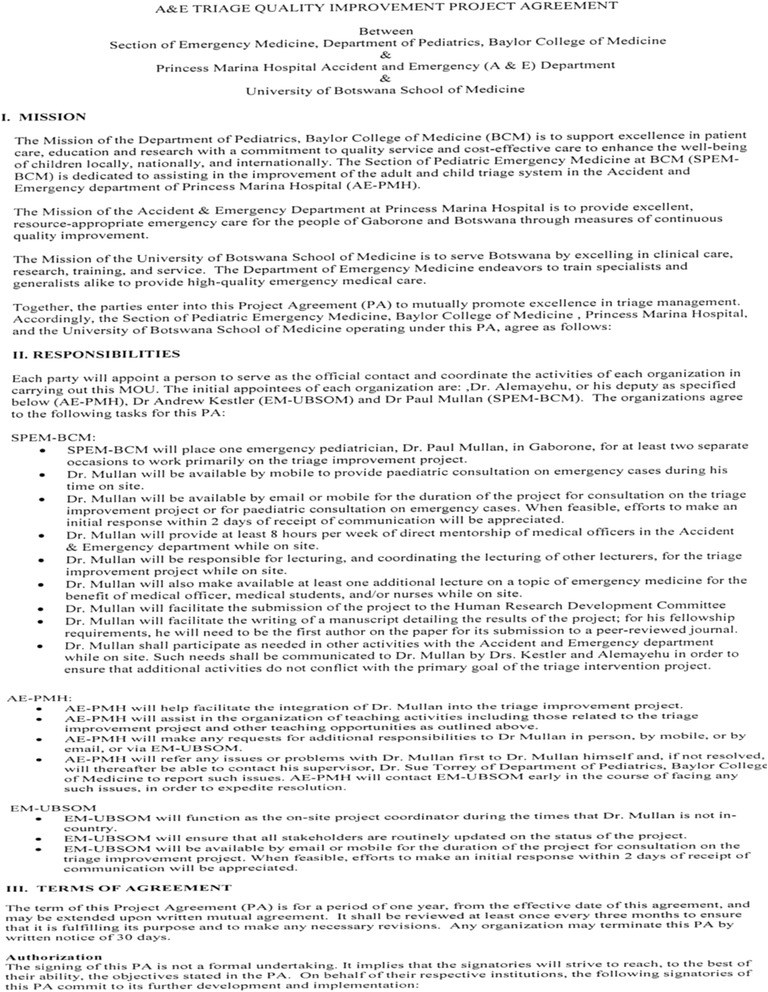
Example of a memorandum of understanding

Partnerships with local institutions, hospitals, or academic facilities are a necessary foundation to successful global health research projects. In creating a global health project, ideally, the first priority is to identify a local partner or institution that will serve as the “Local Champion” for the proposed project. This is often done over time, building a partnership individually or with a group. This long-term partnership is key to success as evidenced in the above-illustrated cases. Secondly, the project should be mutually beneficial to all parties involved and the identified partner should be equally committed and interested in the research project. In case two, the local team approached PM, highlighting their need and commitment. In academic settings, identifying partner priorities for research and offering authorship roles can be a motivating factor. Finally, delineating clear roles and expectations of both partners involved through memorandums of agreement/understanding (MOA/MOU) (Fig. 1) will provide concrete guidance for the project partnership. This could include responsibilities such as funding, housing, teaching, and authorship. Ideally, if these three key concepts are present then a successful partnership and a global health project are feasible [8, 30–32]. Table 5 presents priorities and pitfalls in creating durable and productive partnerships.

**Table 5:** Priorities and pitfalls in creating durable and productive partnerships

Priorities	Pitfalls
Mutual benefit	Not understanding partner priorities or imposing external priorities
Mutual investment/involvement	Not engaging key stakeholders as a voice in the “decision making” process
Identify funding and mobilization of resources	Wasting recipient hospital or country resources
Minimize inequity	Unilateral interest
Find a local champion	Not understanding or identifying unique barriers to specific champions and/or research in partnering countries
Promote local ownership	“One size fits all mentality”
Partner capacity building	Sustainability of programs or projects
Relationship building	Lack of trust and transparency in partnerships
Understanding the political and legal landscape	Not discussing these issues with local partner and institution

### Logistics

The successful completion of an international project depends largely on logistical planning of timelines, budget, implementation, and manuscript publishing.

Timelines should include time for local partner identification, study design and development, data collection, and manuscript writing and submission. Careful planning should account for differing cultures and potential delays, such as availability of local partners and obtaining local IRB approval.

Expenses should be projected early in the project and funding options explored. Oftentimes, the pilot study may have to be funded by personal means. However, a partial list of potential funding agencies for clinicians is available in Table 6. Recently, Hansoti et al. described the potential funding opportunities available specifically for emergency medicine physicians working in international settings. This paper helps the reader navigate the complicated world of grants from seed to federal grants [33]. In addition, gaining the support of the principal investigator’s home institution can be critical in funding time and travel expenses, as well as finding home institution global research funds.

**Table 6:** Global Health Research Funding opportunities for clinicians

American Academic of Pediatrics, Section on International Child Health (SOICH) [12] Fogarty International Clinical Research Scholars & Fellow Program Support Center [44] USAID Global Health Fellows II [45] Fogarty International Center: • NIH funding opportunities [46] • Non-NIH funding opportunities for faculty [47] Bill & Melinda Gates Foundation. Grant opportunities [48] Grand Challenges in Global Health. Grant opportunities [49] Pivot™ [50] National Science Foundation. Active funding opportunities [51] Office of International Affairs. Global Health Initiative––funding [52] Grand challenges in global health. Rewarding innovative ideas [53] Center for global health. The University of Chicago. Funding agencies for global health opportunities [54] American Nurses Association. Opportunities for research funding [55]	

In planning for project implementation, health and safety travel preparedness should not be ignored. Research trip requirements can be found by visiting the public domains of the Centers for Disease Control and Prevention [34], the World Health Organization [35], the home country’s embassy website in the partner country as well as the partnering country’s embassy websites. Planning for the visiting members of the team with clear roles and check-in mechanisms is critical for both the safety and functionality of the team.

## Limitations

First, this checklist is limited by the fact that it is focused on clinicians and may not benefit other researchers involved in global health research such as public health providers. However, this is a deliberate choice, as it is a gap in the published literature and based on our prior experience an invaluable tool for our clinician colleagues.

Moreover, this checklist requires validation, which we will be undertaking.

## Conclusion

**Fig. 2:**
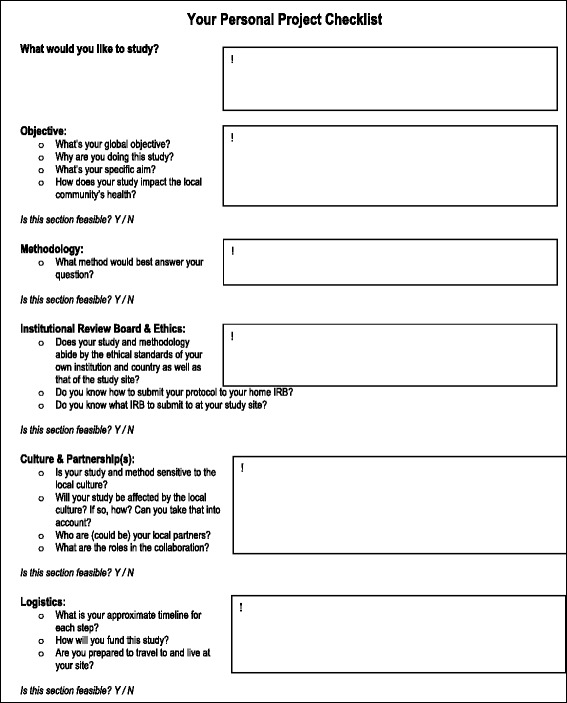
Personal checklist

We have delineated a comprehensive *Global Health Research Checklist for clinicians*, consisting of five sections that we have deemed necessary for a successful project. We believe that the checklist presented above is a valuable tool to plan and assess the feasibility of global health research projects. We have highlighted specific areas a clinician researcher should address when embarking on a global health research project. Figure 2 reproduces the checklist with specific questions to be used by the clinician researcher. Having developed this checklist based on experience and currently available literature, our future aim is to validate it.
